# Supplementary material for: Determinants of neonatal mortality among hospitalised neonates with sepsis at Queen Elizabeth Central Hospital, Blantyre, Malawi: A mixed-methods study
Source: PLOS Glob Public Health. 2024 Dec 18;4(12):e0004059. doi: 10.1371/journal.pgph.0004059 (PMC11654934; doi:10.1371/journal.pgph.0004059)
Supplement: S1 Appendix — (PDF) [file pgph.0004059.s001.pdf]

Appendix 3: Key Informant/In-depth Interview guide for Neonatal Care unit Health workers (English Version)

Study Title: Determinants of neonatal mortality among hospitalized neonates with sepsis in the neonatal care unit at Queen Elizabeth Central Hospital, Blantyre in Malawi

Introduction

My name is Lucky Mangwiro, a student at KUHeS studying Masters of Science in Global Health Implementation and currently in the final year. We are conducting a research study on the determinants of neonatal mortality among hospitalized neonates with sepsis in the neonatal care unit at Queen Elizabeth Central hospital in Malawi, in which you are requested to take part. Please feel free to participate in this study. I will ask you some few questions. Feel free to answer to the best of your knowledge and honestly. It is also your right to withdraw from the study at any time if you feel to do so.

The interview can take less than 30minutes to be completed.

(After introducing myself, explaining the purpose of the interview, seeking consent and finding a location that will make the interviewee comfortable) Facility/Ward level (Target: Doctors, clinical officers, Nurses, ward in charges, Matrons in the neonatal care units)

Interviewee Code:

Facility Name:

Date Collected:

Location:

Time:

Data Collector:

1. Can you tell me about yourself? What is your role (s) in this facility/unit?
   Probe: (age, cadre, qualifications, years of work experience, period worked in the neonatal care unit, medical care? nursing care, any formal trainings on management of neonatal sepsis?
2. What do you understand about neonatal sepsis?
   Probe: causes, signs, risk factors, mode of diagnosis, complications. What about the way people handle neonatal sepsis cases–in the ward, within the hospital? What is your experiences in managing neonatal sepsis? How many sepsis cases in a month and how many neonatal deaths occurred due to sepsis in a month at this facility?
3. What are the risk factors that contribute to neonatal deaths with sepsis at this facility?
4. What are some of the infection prevention measures that are followed in this unit?
   Probe: How do you do it? How often? Chlohexidine cord care done to the neonates at this facility.

5. Does the ward have all the necessary resources to be used in managing sepsis cases?

Probe: examples of resources that you use to manage the sepsis cases at this facility

6. What are the challenges faced when it comes to management of neonatal sepsis at this facility?

Probe: Explain what challenges faced in the management of neonatal sepsis? How are decisions about management of neonates with sepsis made? What is the role of the hospital management team? How do you engage other staff in the management of neonates with sepsis?

7. What efforts/other interventions that can be put in place by the hospital management team, regarding the best practices of hospital management of risk factors of neonatal sepsis to reduce the burden of morbidity and mortality at this facility.

Probe: Has the hospital had standard operating procedures, policies and guidelines in the management of neonatal sepsis/availability of reference materials in management of neonatal sepsis? How do you access guidelines for managing neonatal sepsis? At discharge, describe arrangements that are made for continued care like follow-up visits/reviews?

8. What recommendations might have strengthened the best hospital management practices to improve the care of neonates with sepsis at national and facility level?

Probe: *what improvements or changes to achieve the reduction of neonatal deaths due to sepsis in your facility? How can you best support facilities implement these recommendations?*

9. Are there any family, community– societal barriers to the management of neonatal sepsis at this facility that you have experienced?

Thank the interviewee for their time and inform them that they will be made aware of findings.
