# Supplementary material for: Determinants of neonatal mortality among hospitalised neonates with sepsis at Queen Elizabeth Central Hospital, Blantyre, Malawi: A mixed-methods study
Source: PLOS Glob Public Health. 2024 Dec 18;4(12):e0004059. doi: 10.1371/journal.pgph.0004059 (PMC11654934; doi:10.1371/journal.pgph.0004059)
Supplement: Attachment — Submitted filename: Responses to the Reviewers.pdf [file pgph.0004059.s003.pdf]

KAMUZU UNIVERSITY
OF HEALTH SCIENCES

RESPONSES TO THE REVIEWERS

We have incorporated all the reviewers' comments and pleased to submit our revised manuscript with tracked changes as well as detailed responses. Both tracked changes and a clean version manuscript are attached. The responses to the reviewer comments are outlined below;

Reviewer #1:
This is overall a very well thought out and executed study and a really useful first step in thinking about improving neonatal sepsis care at your hospital. A couple of clarifications are needed in the article.

1. In the methods, how was sepsis defined? Did you use a specific tool, local guidelines (if so, what were the guidelines)?

   Response: We defined sepsis as a diagnosis of clinically suspected neonatal sepsis with confirmed blood culture positive between 1 January to 31 December 2021 among neonates aged 0-28 days admitted at QECH NCU. This is in the methods section under the study design section (L93-95) on page 5. We did not use any specific tool but we used the local guidelines within the unit that are followed to define neonatal sepsis with reference to the Care for Infant and Newborn in Malawi (COIN) manual in session 15 on page 231 states that sepsis is a clinical syndrome of systemic illness accompanied by septicemia (bacteria in the blood).

2. In the methods, were interviews structured, semi-structured? How were the questions developed? It is fine if the questions were developed based on researcher/local expert consensus, but would be good to know (may be useful to share the questions in an appendix as well)

   Response: In the methods we have clarified that the interviews were conducted using a semi-structured interview guide. The questions were developed and informed with an analytical framework for the study of child survival in developing countries by Mosley et al that was modified to the conceptual framework of factors associated with neonatal mortality in the Malawian setting based on the available information in the sampled facility. The variables for the risk factors were categorized into three: distal factors included socio-economic and demographic variables; intermediate factors included maternal reproductive and health delivery factors; and proximal factors included neonatal factors. Refer to the Recruitment, sampling and data collection section on qualitative sampling section where there was a narration of information of the interview guide (L150-157) on page 7-8, abstract section (L12) on page1 and attached interview guide labelled as S1appendix 1 (L684) on page 40.

Mahatma Ghandi Road • P/Bag 360, Chichiri, Blantyre 3 • Tel: +2651 810 911 / +2651 811 286 • Email: registrar@kuhes.ac.mw
www.kuhes.ac.mw @KuHeS_mw

Methods: *grammar check: page 9 line 158 "the data was inductive and deductive coding". It should probably say Data was analysed using inductive and deductive coding.

Response: The sentence grammar was corrected. It now states that data were analysed using inductive and deductive coding. Refer to the methods section under qualitative data analysis (L195) on page 9.

Discussion: several themes were mentioned in the discussion as important factors based on the qualitative analysis-was there one in particular that stood out or one that you would preferentially target?

Response: Several themes were indeed mentioned in the discussion section on the qualitative analysis. The theme that stood out is the delays in neonatal care service cascades such as delayed referral, delayed blood culture results, and delayed treatment. Refer to discussion section L438-439 on page 28.

Line 434 in discussion, you talk about data from Pakistan-is there data from sub-Saharan Africa to suggest that there is sub-standard care being provided in the NICUs (there are a few papers out there)? Did you notice this in your facility? I think throughout your discussion it is important to highlight that while your findings are consistent with other literature (which is valuable to note), it is also specific to your institution. Quality of care provision can vary greatly from one institution to another just within the same district, so it can be abit of an overgeneralization to encompass all LMICs. So, I would be cautious while making the point (while many global concerns may be the same, specific institutions have different outcomes).

Response: We reviewed and revised the sentences by not overgeneralizing. LMICs has been deleted and explained that it was a study that was done in Pakistan that indicated the substandard care but our study only found that the deliveries are being conducted in the health facility by the unqualified skilled personnel. Furthermore, we have been cautious by discussing our findings that they were specific to our institution considering that institutions vary. Refer to the discussion section L491-496 on page 30.

Reviewer #2:

Abstract well written, clearly shows the aim and objectives of the research, the methods and conclusion. The use of mixed methods was also good to explore both quantitative and qualitative figures. The writer maybe should have written how they got to 237 folders for the study. Was there a power calculation that was done?

Response: The explanation of how we got the 237 folders has been clearly explained in the revised manuscript "The sample size was calculated using

Cochran's formula: $n = z^2pq/e^2$, where n was the minimum sample size needed, p was the estimated prevalence rate of neonatal mortality due to sepsis in Malawi (0.19), e was the error rate (0.05), q was 1-p, and z was the standard variable corresponding to the 95% confidence interval (1.96). The calculation yielded a sample size of 236.48, which was rounded up to 237. Power calculation was not done. Refer to the revised manuscript in the methods section under recruitment, sampling, and data collection in the subsection of quantitative data L110-115 page 6.

Reviewer #3:

Suggested comments and revisions;

Line 32, the reference for the literature starts from 24, please make sure it starts from one

Response: The references have been updated. Refer to the revised manuscript with references starting from one to 28 and references section (L579-683) from pages 34-39.

Line 63-66, Long sentence, what does 12.6% for? State clearly

Response: The 12.6% has been stated clearly in the revised manuscript. The 12.6% refers to the prevalence of neonatal sepsis among newborns admitted to the Neonatal Care Unit (NCU) at Queen Elizabeth Central Hospital (QECH) in Malawi from January 1, 2021, to December 31, 2021. We have clarified it as follows: out of all the newborns admitted to the NCU at QECH in Malawi during 2021, 12.6% were diagnosed with neonatal sepsis. Refer to the introduction section (L67-69) on page 4.

Line 73, study setting: include data on admission rate or number of QECH

Response: We have included the admission data for the study setting. In 2021, the NCU admitted a total of 3,717 newborns, with monthly admissions ranging from 170 (March) to 377 (November). The mortality rates varied significantly throughout the year, from a low of 1.2% in March to a high of 20.9% in December, with a total of 470 deaths recorded for the year. Refer to methods under the subsection of study setting (L84-89) from pages 4-5.

Line 103, Cochran's formula is for sample size calculation. Clearly state the sample size determined and the sampling procedure

Response: The sample size determination and sampling procedure has been clarified as follows: "This sample size was calculated using Cochran's formula: $n = z^2pq/e^2$, where n was the minimum sample size needed, p was the estimated prevalence rate of neonatal mortality due to sepsis in Malawi (0.19), e was the error rate (0.05), q was 1-p, and z was the standard variable corresponding to the 95% confidence interval (1.96). The calculation yielded a sample size of 236.48, which was rounded up to 237. To select these records, we used a simple random sampling technique. Numbers were assigned to eligible participants in the sampling frame, and the RANDBETWEEN (1, N) function in Microsoft

Excel was used to randomly select the participants' records for inclusion in the study". Refer to the revised manuscript in the methods section under recruitment, sampling, and data collection in the subsection of quantitative data L110-119 page 6.

Line 151, what app was used to analyse the qualitative data?

Response: The qualitative data were manually analysed and used a thematic approach. Refer to the abstract section (L15) on page 1 and the data analysis section under the subsection of qualitative data (L189-203) on page 9.

Line 178, IQR

Response: We have spelled out IQR in full before the abbreviation. Refer to the revised manuscript in the section of data analysis under a subsection of quantitative data analysis Ll80 on page 9 and the results section under a subsection of quantitative results L217 on page 10.

Line 183, Table 1: I suggest if you could merge frequencies below 10 unless the data need to be displayed separately

Response: We reviewed and merged the frequencies below 10. Here are the categories we have combined in the revised table 1: mother's residence: combined all locations other than Blantyre into "other", maternal chronic conditions: combined "Diabetes" and "other" into a single "other" category, VDRL status: combined "Positive" and "unknown" into "unknown/Positive", birth location: combined "In transit", "home delivery", and "not recorded" into "other/not recorded", reason for admission: combined "temp too high", "temp too low", "congenital malformation", "convulsions", "other maternal conditions", and "other" into a single "other" category, mode of delivery: combined "Vaginal breech", "Vacuum extraction", and "unknown" into "other/unknown". Refer to table 1 titled Characteristics of mothers and newborns admitted with sepsis to QECH on results section under subsection of quantitative data (L226-229) from pages 13-16.

Line 219-222, The connection between health system factors and maternal factor is not clear. Instead of health system factor, it is more of maternal health related factors

Response: Note that lines 219-222 explain maternal health-related factors, not the health systems factors. Refer to the results section in the qualitative data subsection of maternal factors under maternal health-related factors(L266-282) on pages 20-21 while the health system factors were explained in the results section under the qualitative data subsection of health system factors refer to (L319-422) on pages 23- 27.

Line 237, some of the lists under behavioral factors are not related to the mothers' behaviour like filthy delivery location, contaminate equipment

Response: We reviewed and noted that some of the listed factors under behavioural factors were not related and hence deleted. Refer to the result section under qualitative data on maternal behavioural factors (L283-285) on page 21.

Line 259, state BBAs before abbreviations

Response: We have spelt BBA in full before abbreviating it. Refer to the revised manuscript in the section of results under qualitative data on the neonatal factors section (L305-306) on page 22.

Line 275, what information?

Response: Thank you for the comment. The "what information" refers to the lack of training for healthcare workers as one of the health system factors contributing to neonatal deaths related to sepsis. Refer to the results section under qualitative data on the health systems factors section (L321-323) on page 23.

Line 394-398, there are new qualitative results that hasn't been reported in result section, include in the results or exclude from the discussion

Response: This has been addressed by excluding the new qualitative results reported in the discussion section since they did not come out clearly in the qualitative data result section. Refer to the discussion section (L445-447) on page 28.

Line 408-412, result on ANC attendance not shown

Response: Indeed, results on ANC attendance are not shown in the result section and discussion section. The sentences on literature for other studies on ANC attendance have been deleted and the sentences have been revised. Information on ANC attendance was not clearly stated in either quantitative or qualitative data but rather ANC attendance was mentioned in the discussion section as one of the recommendations, to timely identify risk factors, early diagnose and treat maternal infections and other health problems during pregnancy might help to identify possible interventions to reduce the risk factors of adverse birth outcomes, hence reduce mortality. Refer to the discussion section (L455-465) on page 29.

We appreciate the reviewers for their guidance and constructive feedback.

Thank you for your consideration.
