# Supplementary material for: Determinants of neonatal mortality among hospitalised neonates with sepsis at Queen Elizabeth Central Hospital, Blantyre, Malawi: A mixed-methods study
Source: PLOS Glob Public Health. 2024 Dec 18;4(12):e0004059. doi: 10.1371/journal.pgph.0004059 (PMC11654934; doi:10.1371/journal.pgph.0004059)
Supplement: Attachment — Submitted filename: Review notes PGPH-D-24-00266.docx [file pgph.0004059.s002.docx]

Manuscript Number: PGPH-D-24-00266

Full Title: Determinants of neonatal mortality among hospitalized neonates with sepsis at Queen Elizabeth Central Hospital, Blantyre, Malawi: A mixed-methods study

Reviewer: Gadise Bekele Regassa

Suggest comments and revisions

| Line number | Comment/suggested revision | Remark |
| --- | --- | --- |
| 32 | The reference for the literature starts from 24, please make sure it starts from one |  |
| 63-66 | Long sentence, what does 12.6% for? State clearly |  |
| 73 | Study setting: include data on admission rate or number of QECH |  |
| 103 | Cochran’s formula is for sample size calculation. Clearly state the sample size determined and the sampling procedure. |  |
| 151 | What app is used to analyzed the qualitative data? |  |
| 178 | IQR |  |
| 183 | Table 1: I suggest if you could merge frequencies below 10 unless the data need to be displayed separately |  |
| 219-222 | The connection between health system factors and maternal factor is not clear. Instead of health system factor, it is more of maternal health related factors. |  |
| 237 | Some of the lists under behavioral factors are not related with the mother’s behavior like filthy delivery location, contaminated equipment |  |
| 259 | State BBAs before abbreviations. |  |
| 275 | What information? |  |
| 394-398 | There are new qualitative results that hasn’t been reported in result section, include in results or exclude from discussion |  |
| 408-412 | Result on ANC attendance not shown |  |
